# Determinants of complex regional pain syndrome type I in patients with scaphoid waist fracture- a multicenter prospective observational study

**DOI:** 10.1186/s12891-021-04977-0

**Published:** 2022-01-05

**Authors:** Hao Gong, Gang Zhao, Yuzhou Liu, Zhengfeng Lu

**Affiliations:** 1 Department of Hand Surgery, Wuxi Ninth People’s Hospital, Wuxi, Jiangsu China; 2 Department of Medicine, Soochow University, Suzhou, China

**Keywords:** Scaphoid waist fracture, Complex regional pain syndrome type I, Pain, Risk factors

## Abstract

**Background:**

The aim of this prospective study was to assess the incidence of complex regional pain syndrome type I (CRPS I) in patients with scaphoid waist fracture and to explore associated factors.

**Methods:**

This was a multicenter, prospective observational study. Demographic, imaging indicators and clinical data were collected before the conservative treatment of scaphoid waist fracture patients. The occurrence of CRPS I and pain condition were the main outcomes. To explore the factors associated with CRPS I, multivariate logistic regression model was used.

**Results:**

A total of 493 scaphoid waist fracture participants undergoing conservative treatment were recruited for this study. The incidence of CRPS I was 20% (*n* = 87). The average time between injury and the onset of CRPS I was 6.7 ± 2.1 weeks. Multivariable logistic regression analysis revealed that female sex (odds ratio (OR): 1.669; 95% confidence interval (CI): 1.189–2.338), diabetes mellitus (OR: 3.206; 95% CI: 2.284–4.492), and severe pain condition before treatment (visual analog scale (VAS) score more than 4 cm) (OR: 27.966; 95% CI: 19.924–39.187) were independently associated with CRPS I.

**Conclusions:**

Patients suffering from scaphoid waist fracture may be at a higher risk of CRPS I, especially in women with diabetes mellitus who report severe pain before treatment. Early screening and regular follow up evaluation are recommended in these patients.

## Background

Complex regional pain syndrome type I is a chronic pain condition, which characterized by spontaneous abnormal pain intensity and duration. In two population-based studies performed in North America [1] and Europe [2], the incidence of CRPS I was 5.5 and 26.2 per 100,000 person-years, respectively. The common symptoms of CRPS I include abnormal sensation, vasomotor edema changes, and motor dysfunction [3]. The clinical signs include four categories: the evidence of sensory; vasomotor; edema and trophic. The diagnosis of CRPS I involves the presence of at least two clinical signs included in the four categories and at least three symptoms in its four categories [4]. Previous studies [5] reported that trauma was the most common events of CRPS I and the athletes can be easily affected by CRPS I because of exposure to traumatic or overuse injuries [6]. The causes of CRPS I are unclear. Evans et.al [4] reported that an excess of afferential input related to the damaged tissue could start a chain of activation involving sympathetic neurons. The activity of sympathetic postganglionic fibers could produce spasms of the arteries and therefore ischemia with increased capillary filtration pressure, edema and swelling. Generally speaking, comprehensive approach maybe more suitable for CRPS I, including pharmacological therapy, physical therapy, therapeutic exercise, and neurorehabilitation, psychological and educational interventions [4]. The timing of the pharmacological intervention is important for treatment outcomes. Prednisolone might be useful for the treatment of CRPS and vitamin C could work as a preventive strategy [7].

According to the authors’ clinical experience, CRPS I may develop after scaphoid fractures, which was also be reported in previous studies [8–10]. Distal radius fracture was the most common induced event of CRPS I and it was also the most commonly encountered associated injury of scaphoid fracture [11]. However, few studies have focused on the incidence of CRPS I in scaphoid fractures patients, most articles mainly focus on the wrist fracture, which was includes multiple fracture types [10]. In addition, exploring the risk factors for CRPS I is important, which helps clinicians establish prevention measures and alleviate the discomfort of patients.

The aim of the study was to assess the incidence of CRPS I in patients with scaphoid waist fractures and to explore the associated factors.

## Methods

### Study design

This was a multicenter, prospective observational study that was approved by the ethics board of the four participating institutions. Signed informed consent was obtained from all patients.

### Patients

The baseline data were collected before conservative treatment, and included demographic and imaging indicators and clinical data. The demographic data included age, sex, dominant hand, injured side, body mass index, education, job status, socioeconomic status, days between injury and casting, type of trauma, fracture type, current tobacco use, and diseases, including hypertension and diabetes mellitus. According to the image data, imaging indicators were measured at each participle, which included the maximum displacement, scapholunate angle, radiolunate angle, carpal height index ratio, and lateral intrascaphoid angle [12].

From August 2016 to November 2019, scaphoid waist fracture patients were recruited from the orthopedics department of four level I trauma centers in China. Scaphoid waist fracture is primarily diagnosed by imaging studies, including radiographs and CT scans. The radiograph exam included five radiographic views: posterior–anterior, lateral, semisupine, semiprone, and elongated scaphoid. All imaging data were collected by different recruiting sites and assessed by the same team, which included one senior radiologist (GZ) and one senior orthopedic doctor (GH). Convenient sampling was used as a sampling method. All patients received standard conservative treatment pathway, which includes cast immobilization, patient education and rehabilitation training [13]. The wrist was cast using the below-elbow cast without inclusion of the thumb in neutral alignment for 10 weeks.

The following inclusion criteria were used: (1) Bicortical scaphoid waist fractures, which were defined as a break in the continuity of both cortices on any radiographic view [14]; (2) nondisplaced or minimally displaced fracture, which was defined as less than 1 mm of gapping or translation between fracture fragments on CT or on any of five radiographic views; (3) The time from injury to hospital patients was less than 2 weeks; and (4) patients underwent conservative treatment. The exclusion criteria were as follows: (1) patients who received surgical treatment; (2) patients with concomitant injuries of the upper limb; (3) age ≤ 18 years; (4) patients with a history of CRPS I and other chronic pain conditions because these patients have a higher incidence of recurrence [15]; (5) patients who refused to participate in the study; (6) during the treatment, patients suffer complications, such as delayed- or nonunion, and other conditions require secondary intervention; and (7) pregnant and lactating women.

#### Primary outcome measures

**Fig. 1:**
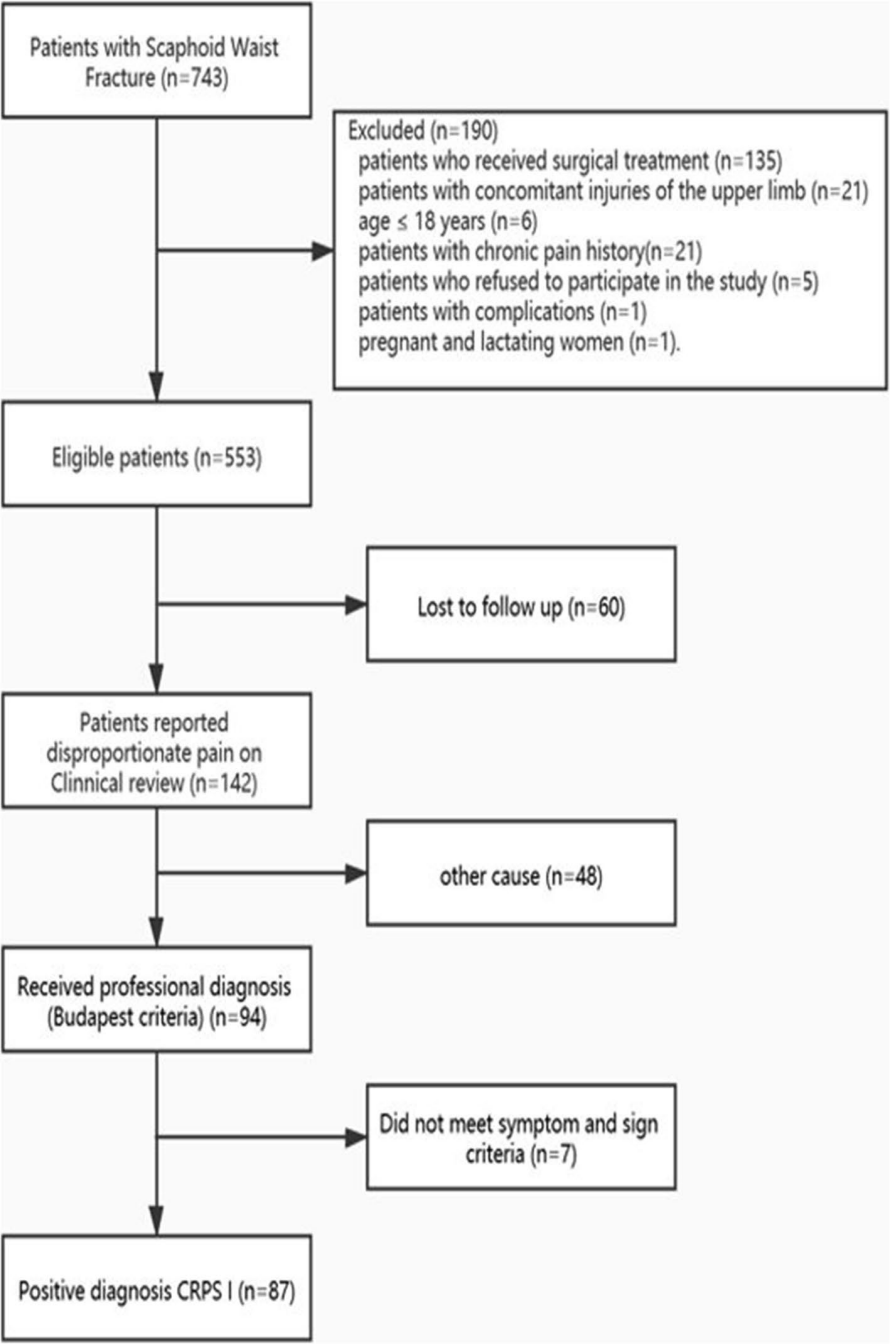
Flow chart of patient inclusion in the present study

The primary outcome of this study was the occurrence of CRPS I. The patients were followed-up every week during the period of cast immobilization and the first 3 months after cast removal and at the 6-, 9- and 12-month follow-ups. A 10 cm VAS scale (0 = no pain, 10 = total pain) was used by a pain specialist (YL) at each follow-up to assess the patient’s pain level. Few scaphoid waist fracture patients reported greater pain (VAS > 4 cm) within 1 year after treatment in previous studies [16, 17]. Therefore, 4 cm was defined as the cutoff score of this study. For patients who present disproportionate pain in the operated limb, we initially attempted to exclude other potential causes, and then we made the diagnosis of CRPS I according to Budapest criteria [18]. The specific process is shown in Fig. 1.

#### Data collection techniques

The clinical data included pain condition, wrist function, quality of life, psychological condition, and radiological measurements. Baseline pain condition was also assessed using the VAS. A dynamometer (Zhengyi Company, Tianjin, China) was used to measure the wrist function, including the range of motion and grip strength. The ranges of motion include flexion, extension, pronation, supination, ulnar deviation and radial deviation. The grip strength is presented as the percentage compared to the healthy side. We also assessed wrist function using the Quick Disabilities of the Arm, Shoulder, and Hand (QuickDASH) questionnaire [19, 20] (0 = no disability, and 100 = total disability). The patient-rated wrist evaluation score (PRWE) score was used to calculate the wrist pain and disability, and contained 3 parts and 15 items (0 = no disability and 100 = maximum loss of function and marked pain). The Mayo modified wrist score was calculated preoperatively in each patient [21]. The Chinese version of the Short-Form Health Survey (SF-12) was used to evaluate quality of life [22]. The patient’s psychological conditions were evaluated using the Hospital Anxiety and Depression Scale (HADS) (21 items) [23], which included two subscales (anxiety and depression) and 14 questions. Eight points were defined as the cutoff scores of depression and anxiety, which divided the patients in to abnormal cases and normal cases.

#### Statistical analyses

We determined the sample size according to the number of variables. The lower limit of the number of included individuals was at least 10 times the number of events per variable [24]. In this study, 32 variables were included in multivariate analysis. Therefore, 320 patients were required to be enrolled in the study. Considering a possible dropout of 20%, the recruitment target was 88 participants.

Statistical analyses were performed using Statistical Package for the Social Sciences (SPSS, version 25, Chicago, IL). All reported *P* -values were two-tailed. Fisher’s exact test and t-test were conducted as univariate logistic regression analyses. Variables with statistically significant results (*P* < 0.1) in univariate analysis were included in multivariate analysis (Backword-Wald) to identify the variables that were independently associated with CRPS I. The results were expressed as ORs and 95% CIs. Differences were considered statistically significant at *p* < 0.05.

## Results

A total of 743 participants were screened for eligibility, of whom 190 (33.6%) patients were excluded. 553 participants were recruited in this study. A total of 493 (89%) patients, including 295 male and 198 female patients, completed the 1-year follow-up. The mean age was 50.3 ± 4.2 years. The mean time interval from injury to conservative treatment was 3 days. The average time between injury and the onset of CRPS I was 6.7 ± 2.1 weeks. In this study, the results of the Budapest criteria demonstrated that 87 (20%) patients presented with CRPS I.

The demographic and clinical characteristics are summarized in Tables 1 and 2. The factors associated with CRPS I were gender (*p* = 0.031), type of trauma (*p* = 0.035), diabetes mellitus (*p* = 0.005), pain condition before treatment (*p* = 0.001), anxiety (*p* < 0.001), SF-12 mental points (*p* < 0.001), and PRWE pain points (*p* = 0.003). These parameters were included in multivariable logistic analysis to identify the independently associated factors of developing CRPS I in scaphoid waist fracture. Female sex (OR: 1.669; 95% CI: 1.189–2.338), diabetes mellitus (OR: 3.206; 95% CI: 2.284–4.492), severe pain condition before treatment (VAS more than 4 cm) (OR: 27.966; 95% CI: 2.323–6.151) were independently associated with CRPS I (Table 3).

**Table 1:** Demographic characteristics of study sample

Characteristics	Patients With CRPS I (*n* = 87)	Patients Without CRPS I (*n* = 406)	*P*
**Age (year)**	51.5 ± 19.2	49.7 ± 18.8	0.420
**Gender, n (%)**			
**Male**	61 (70)	234 (58)	
**Female**	26 (30)	172 (42)	0.031*
**Dominant hand, n (%)**			
**Left**	8 (9)	44 (11)	
**Right**	79 (91)	362 (89)	0.651
**Injured side**			
**Dominant**	74 (85)	348 (86)	
**Non-dominant**	13 (15)	58 (14)	0.874
**Body Mass Index (kg/m**^**2**^**)**	20.7 ± 2.9	20.4 ± 3.0	0.395
**Education, n (%)**			
**University**	55 (64)	288 (71)	
**Primary and middle**	30 (34)	112 (28)	
**Illiterate**	2 (2)	6 (1)	0.351
**Job status, n (%)**			
**Unemployed**	23 (26)	102 (25)	
**Employed**	64 (74)	304 (80)	0.798
**Socioeconomic status, n (%)**			
**High**	19 (22)	75 (18)	
**Medium**	61 (70)	287 (71)	
**Low**	7 (8)	44 (11)	0.616
**Time from injure to casting**	3.1 ± 0.8	3.0 ± 1.1	0.422
**Type of trauma, n (%)**			
**Twisting**	5 (6)	36 (9)	
**Fall**	42 (48)	284 (70)	
**Punch**	20 (23)	46 (11)	
**MVA**	12 (14)	42 (10)	
**Uncertain**	8 (9)	18 (4)	0.002*
**Fracture type, n (%)**			
**Waist**	78 (90)	363 (89)	
**Distal**	9 (10)	43 (11)	0.946
**Current tobacco use, n (%)**	25 (29)	118 (29)	0.904
**Diseases**			
**Hypertension, n (%)**	19 (22)	91 (22)	0.907
**Diabetes mellitus, n (%)**	15 (17)	`132 (33)	0.005*

* *P* < 0.05

**Table 2:** Clinical characteristics of study sample

Characteristics	Patients with CRPS I (*n* = 87)	Patients without CRPS I (*n* = 406)	*P*
**Pain condition before treatment (VAS, cm)**	6.2 ± 16.5	5.7 ± 1.3	0.001*
**Grip strength (kg)**	37.2 ± 18.4	39.5 ± 16.9	0.257
**Grip strength (% value on healthy side)**	65% (50 to 69)	61 (52 to 64)	0.162
**Quick DASH (points)**	74.1 ± 1.9	74.6 ± 2.3	0.059
**Maximum displacement (mm)**	1.1 ± 0.6	1.2 ± 0.4	0.056
**Mayo Modified Wrist Score (points)**	87.6 ± 18.2	91.5 ± 17.6	0.063
**Wrist range of motion (°)**			
**Flexion**	78.2 ± 19.3	77.8 ± 18.5	0.856
**Extension**	81.4 ± 12.3	80.4 ± 12.7	0.103
**Pronation**	80.2 ± 17.5	80.8 ± 16.7	0.763
**Supination**	86.5 ± 12.9	85.4 ± 11.2	0.419
**Ulnar deviation**	32.1 ± 8.2	31.9 ± 7.8	0.830
**Radial deviation**	18.8 ± 5.7	19.3 ± 6.5	0.507
**HADS (points)**			
**Depression**	7.4 ± 3.1	6.9 ± 2.5	0.106
**Anxiety**	7.1 ± 3.8	6.0 ± 2.2	< 0.001*
**SF-12 (points)**			
**Physical**	48.4 ± 3.6	49.1 ± 7.2	0.378
**Mental**	42.9 ± 4.3	43.4 ± 4.8	< 0.001*
**Mayo Modified Wrist Score (points)**	87.1 ± 18.9	86.8 ± 16.3	0.880
**Radiographic measures (°)**			
**Scapholunate angle**	81° (71 to 87°)	81° (67 to 88°)	0.342
**Radiolunate angle**	29° (22 to 33°)	30° (23 to 30°)	0.416
**Carpal height index**	0.45 (0.43 to 0.50)	0.44 (0.42 to 0.50)	0.234
**Lateral intrascaphoid angle**	68° (59 to 74°)	68° (60° to 74°)	0.146
**PRWE (points)**			
**Pain**	19.3 ± 1.3	18.9 ± 1.4	0.003*
**Function**	16.7 ± 2.3	17.1 ± 1.7	0.063
**Total**	35.1 ± 2.1	34.9 ± 2.5	0.487

*VAS* 100-mm visual analogue scale, *Quick DASH* Quick Disabilities of the Arm, Shoulder, and Hand, *PRWE* Patient-rated wrist evaluation score

Non-dominant hand values increased by 5%

* *P* < 0.05

**Table 3:** Logistic regression for variables predictive factors of occurrence of CRPS I

Variable	β	Odds ratio		95% CI	*P* value
**Gender (female)**	0.512	1.669		1.189–2.338	0.023*
**Type of trauma (twisting)**	0.321	1.379		0.982–1.932	0.112
**Diabetes mellitus**	1.165	3.206		2.284–4.492	0.002*
**Pain condition before treatment (> 4 cm)**	1.331	3.785		2.323–6.151	0.021*
**Anxiety**	1.293	3.644		2.596–5.106	0 < 0.001
**SF-12**	0.062	1.064		0.623–1.811	0.069
**PRWE**	0.135	1.145		0.670–1.949	0.118

Multivariable logistic analysis was used, * *P* < 0.05

*PRWE* Patient-rated wrist evaluation score

## Discussion

Scaphoid fracture is a frequent and common carpal fracture and accounts for 2 to 7% of all fractures [13]. Young people are the most affected population [13]. However, chronic pain conditions may occur after the treatment, which could potentially disable patients for an extended time. Chronic pain conditions may be caused by some surgical factors, such as nonunion and malunion, but in most chronic pain cases no obvious cause can be found. The presence of CRPS I is one of the major underlying causes of chronic pain conditions [25].

In total, 438 patients were recruited consecutively and completed the 1-year follow-up. The incidence of CRPS I in this study was 20%, which is lower than that in distal radius fracture patients (32.2%) [26]. The disparity of diagnostic criteria could partly explain this difference. Veldman criteria and Budapest Diagnostic Criteria were used in the two studies. The incidence of CRPS1 is greatly affected by the diagnostic criteria of CRPS1.The results of Annemerle Beerthuizen et al. [10] indicate that Budapest criteria are recommended in future studies on CRPS1. In fact, diagnosing CRPS I remains challenging in the absence of a uniform criterion standard. Another possible explanation of this lower incidence is the difference in fracture types. Our studies only included scaphoid waist fracture patients. Previous studies [10] have reported that intra-articular fracture patients seem to have a higher chance of developing CRPS1.

In our study, no significant difference was observed in wrist function of two groups, which could be partly explained by the characteristics of the disease. CRPS is a syndrome characterized by a continuing regional pain which is seemingly disproportionate in time or severity to the trauma events [4]. In this study, the statistically significant results of anxiety and mental aspect of SF12 were observed. High preoperative anxiety levels have been verified as a predictive index of the CRPS I following total knee arthroplasty [27]. Female sex, diabetes mellitus, and severe pain condition before treatment were independent risk factors for the development of CRPS I. Female sex has been identified as the risk factors for CRPS I by many studies [28–31]. Previous studies reported that CRPS I seems to be more prevalent in women, and the incidence of women was of 2–4 times that of men [28–31]. Women, especially postmenopausal women, have low vitamin D levels. Vitamin D deficiency is related to neuropathic pain. Sang-Uk Lee et al. [32] reported that the reason why the women’s incidence of CRPS I is higher than men’s may be that neuropathic pain is associated with the low vitamin D levels frequently found in post-menopausal women. Yesil et al. [33] reported that rheumatoid arthritis patients with serum vitamin D levels below 20 ng/mL exhibit an increased prevalence of neuropathic pain compared with patients with vitamin D values 30 ng/mL or over (5.8:1). Therefore, early screening of pain conditions is vital for female patients.

In our study, diabetes mellitus was independently associated with CRPS I. Many studies have reported that diabetes mellitus is highly related to CRPS occurrence [34, 35]. Pro-inflammatory cytokines, including interleukin -1β, interleukin-6 and tumor necrosis factor-alpha, play an important role in the relationship of diabetes mellitus and CRPS I. Skin biopsy results show that the expression of pro-inflammatory cytokines was significantly higher in the upper extremities of CRPS-affected patients than in those of the unaffected patients [36, 37]. Pro-inflammatory cytokines which was derived from fat cells could lead to the resistance to insulin, while excessive fat cells promote the synthesis and secretion of pro-inflammatory cytokines [38]. In addition, Jong Ho Choi et al. [39] verified that higher HbA1c could be related to higher CRPS prevalence and that uncontrolled blood glucose could increase CRPS occurrence. Among the treatment of CRPS, it has always been a controversial issue whether physical therapy has a role for improving the clinical condition [40]. Diabetes mellitus was the risk factor of CRPS I. Our team believed that appropriate physical therapy can produce certain positive role in the prevention of CRPS I by decreasing the blood glucose [38].

In our study, a severe pain condition before treatment was also a risk factor for the development of CRPS I, which was almost identical to previous studies [10, 41] as reported by Barbara et al. [41] that reported that basal pain sensitivity and modulation vary widely in different patients. The sensitization of the nervous system could partly explain this phenomenon [42, 43]. Therefore, the baseline pain condition of fractures deserves more attention.

There are some potential limitations that should be considered. First, the assessment of psychological conditions (anxiety and depression) is controversial because the HADS is a screening tool rather than a clinical diagnostic tool. Second, convenient sampling was used as the sampling method in this study, which could result in a large random error in the estimated prevalence ratio. Third, the small sample size could reduce the generalizability of this study.

## Conclusions

Patients suffering from scaphoid waist fracture may be at a higher risk of CRPS I, especially in women with diabetes mellitus who report severe pain before treatment. Early screening and regular follow up evaluation are recommended in these patients.

## Data Availability

All of the materials were provided by the Wuxi No.9 People’s Hospital Affiliated to Soochow University, The datasets used and/or analyzed during the current study available from the corresponding author on reasonable request.

## Abbreviations

CRPS I: Complex regional pain syndrome type I
OR: Odds Ratios
VAS: Visual analog scale
PRWE: Patient-rated wrist evaluation score
CI: Confidence interval
QuickDASH: Quick Disabilities of the Arm, Shoulder, and Hand
SF-12: Short-Form Health Survey
HADS: Hospital Anxiety and Depression Scale
